# Should Touch Screen Tablets Be Used to Improve Educational Outcomes in Primary School Children in Developing Countries?

**DOI:** 10.3389/fpsyg.2016.00839

**Published:** 2016-06-06

**Authors:** Paula J. Hubber, Laura A. Outhwaite, Antonie Chigeda, Simon McGrath, Jeremy Hodgen, Nicola J. Pitchford

**Affiliations:** ^1^School of Psychology, University of NottinghamNottingham, UK; ^2^E-Learning Centre, School of Education, Chancellor College, University of MalawiZomba, Malawi; ^3^School of Education, University of NottinghamNottingham, UK

**Keywords:** mathematics, hand-held tablets, ICT, developing countries, intervention, early years, literacy

The 2030 Agenda for Sustainable Development aims to “ensure inclusive and equitable quality education and promote lifelong learning opportunities for all” (United Nations, 2015). Whereas, the principal focus of global education planning since 2000 had been on getting children into schools, sustainable development goal 4 (above) reflects concerns about quality. As improving learning outcomes starts to receive heightened policy focus, it becomes imperative to consider the types of intervention that can be most effective in raising learning outcomes, particularly in settings characterized by poor resourcing and persistent low attainment levels. Here, we consider if touch screen tablets can be used to improve educational outcomes in primary school children in developing countries. We focus on early maths attainment in Malawi as one of the most dramatic examples of the current learning challenge.

## Contextual background

Malawi is in desperate need of educational reform (UNESCO, 2015). Its first democratic elections in 1994 saw education become a core electoral issue, with parties seeking to outbid each other on their educational promises. This resulted in a free primary schooling policy, introduced at very short notice, which got millions of children into school, but ultimately could neither deliver good education nor keep learners in schools. More than 20 years on, primary education in Malawi still suffers from high repetition and drop-out rates, poor supply or lack of essential teaching and learning materials in most schools, and severe shortages of qualified teachers. These factors contribute to poor internal efficiency of the education system, as well as impacting negatively on early grade numeracy acquisition in Malawian pupils, a key foundation skill for later learning. Girls and children with special educational needs are particularly vulnerable in this education system, which results in vast inequalities across pupils.

In 2007, The World Inequality Database on Education reported that only 40% of all primary school children in Malawi attained the minimum learning standards. Despite some improvements in recent years, the low level of primary mathematics attainment in Malawi is a significant and continuing concern. In the 2007 Southern and Eastern Africa Consortium for Monitoring Educational Quality (SACMEQ) survey at Grade 6, Malawi's mean mathematics score of 447 was well below the average of 510 for countries participating in the survey (Milner et al., 2011a). Less than 50% of students reached at least the SACMEQ basic numeracy competency level by the end of primary school. Key factors appear to be the availability of resources, particularly textbooks, and the quality of teaching (Chimombo, 2005; Milner et al., 2011b). This suggests that an early intervention, focused on basic arithmetical skills and concepts, that does not rely too heavily on teacher quality might be an effective way of addressing the challenges faced in primary mathematics in Malawi.

## Could digital education technology (DET) provide an innovative solution?

DET has been used with the aim of raising educational attainment in interventions across high income and developing countries. Despite a great deal of government money being spent on DET (Law et al., 2008), outcomes on learning are mixed even for relatively small-scale and well-designed interventions. A review of 74 DET projects attempting to raise mathematics performance across a variety of countries found modest effect sizes for improvement (Cheung and Slavin, 2013). This may be because the technology has not generally had a strong focus on pupil learning. Several small-scale studies have found positive effects for computer use in schools for mathematics (Banerjee et al., 2007; Räsänen et al., 2009; Praet and Desoete, 2014; Sella et al., 2016) and in other domains, including literacy (Ho and Thukral, 2009; Kaleebu et al., 2013). Probably the most well-known and widespread use of DET in the classroom is the One Laptop Per Child (OLPC) intervention. However, this project has had limited success due to poor implementation and lack of teacher training. In Alabama, US, the programme failed due to lack of Internet access in schools and poor support for repairs (Warschauer and Ames, 2010). In Peru OLPC succeeded in increasing the numbers of computers available to students, but no effects were found for improving mathematics or language skills (Cristia et al., 2012). In Rwanda (Fajebe et al., 2013) and Tanzania (Apiola et al., 2011), problems included a lack of both teacher training and child-directed implementation. Teachers often saw themselves, rather than the pupils, as the primary laptop users. In contrast, recent interventions focused on touch screen tablet technology used directly by pupils have shown promising results.

A review of 23 studies published since 2009, which used touch screen tablet technology for improving academic performance in children aged 5–18 across high income and developing countries, found large positive effect sizes in favor of the technology compared to normal classroom practice for a range of subjects (Haßler et al., 2015). Use of tablet technology also improved mathematics performance in pre-school children in a teacher-led intervention in the US and children became more independent learners (Schacter and Jo, 2016). Berkowitz et al. (2015) also found home use of tablets improved mathematics skills in 5–6 year-old children. Other benefits of touch screen tablets include easier use for young children in respect of motor skills (Cooper, 2005; Donker and Reitsma, 2007; Kucirkova, 2014; Outhwaite et al., under review).

## Unlocking talent through technology

To take advantage of the potential benefits of using touch screen tablets to address the issues of low educational attainment and resources and variable teacher quality in Malawi, the Ministry of Education, Science and Technology in Malawi is implementing a new and innovative mobile technology—“oneclass”—across 68 primary schools in partnership with an international charity, Voluntary Service Overseas (VSO), and onebillion, the UK charity behind the technology. All pupils from standards 1 and 2 in the participating schools will use the interactive apps developed by onebillion as part of their mathematics education.

## Oneclass technology and interactive maths apps

This technology consists of a learning center and a series of interactive, child-centered, maths apps that has been developed by the non-profit education publishers, onebillion, for 3–6 year olds. The maths apps are delivered to individual children through an Apple iPad mini connected to a set of headphones. Designed especially to be easy for schools to implement, the software provides clear instruction through a virtual teacher speaking in the local language, at an age-appropriate level, and guides pupils progressively through a series of activities based on the national curriculum. The learning center is a specially designed classroom equipped with solar power to enable children to use the maths apps throughout the day, even in remote rural regions that are off-grid. Remote monitoring ensures that children are using the maths apps and records their progress as they work through the apps. This information is fed back to their teachers, which enables teachers to direct attention to children that become halted on a particular part of the maths apps, and children who are making slow progress. A solar-powered projector in the learning center allows teachers to work with groups of children on a particular topic and provide additional support to slow learners. Even teachers with little subject knowledge can deliver the maths apps to small groups of pupils, thus optimizing efficiency of teaching time whilst delivering high-quality mathematics education to all children.

## Evidence-base for effectiveness

The maths apps developed by onebillion for oneclass have been trialed in Malawi at the pupil level within one urban primary school (Pitchford, 2015). A Randomized Control Trial (RCT) was conducted with 283 primary school pupils spanning standards 1–3. The intervention ran for 8 weeks. Compared to control children receiving normal practice, significantly higher attainment was shown at post-test by children with the maths apps, on both conceptual knowledge (4% higher attainment, Cohen's *d* = 0.23) and curriculum knowledge (18% higher attainment, Cohen's *d* = 0.75). Girls responded just as well to the mathematics intervention as did boys, demonstrating that this technology could prevent the gender disparity that is currently evident in Malawi education.

Despite a radically different educational and cultural context, many children in the UK also struggle to learn basic mathematical skills. A “tail of underachievement” exists amongst disproportionate groups of underachieving pupils (Tymms and Merrell, 2007, p.13), particularly children from low-income areas (Anders et al., 2012). Yet, comparable results with the onebillion maths apps to those found in Malawi were shown in a small pilot study conducted in a primary school in England. A group of 61 pupils aged 4–5 years used the maths apps for 6 weeks as a supplementary early intervention approach to address mathematics underachievement (Outhwaite et al., under review). Significant learning gains in both conceptual knowledge (5% increase, Cohen's *d* = 0.31) and curriculum knowledge (22% increase, Cohen's *d* = 1.01) were found immediately post-intervention, and further learning gains were shown at 4 months later, at delayed post-test (conceptual knowledge increased a further 5% Cohen's *d* = 0.76 and curriculum knowledge increased a further 6% Cohen's *d* = 1.07). Learning gains were not influenced by the child's income background.

These two studies demonstrate proof of concept of a measureable impact of the oneclass intervention on mathematical attainment in primary school pupils in both Malawi and England. This cross-cultural evidence illustrates generalization of the effectiveness of this intervention across extremely different contexts and highlights the potential for this intervention to have global reach.

## Moving to scale

The programme in Malawi has now moved to a medium scale trial that also sees the introduction of a new series of apps designed by onebillion to support literacy acquisition. To effectively assess scalability, we argue that new research is needed to show measurable impact on standardized assessments of numeracy and literacy and across the curriculum more broadly. Consideration of how primary school teachers cope with pupils with numeracy and literacy skills greater than they are used to is also required, as is the impact this has on pedagogical practice and curriculum development. In addition, consideration of how this technology might be used to raise learning outcomes in pupils in challenging contexts is needed, to ensure the intervention is tailored for all children, regardless of location and wealth. Finally, a cost-benefit analysis is required, to determine if the potential advantages of raising mathematical and literacy standards in primary school children in the long-term outweigh the costs of implementing this technology in a low resourced country.

## Concluding thoughts

It is estimated that 250 million children worldwide do not possess the basic numeracy and literacy skills required to live a healthy and productive life and contribute toward economic growth (UNESCO, 2014). We have identified the possibilities that digital education technologies might have in increasing early learning outcomes in developing countries such as Malawi. We argue that to better understand the efficacy of using touch screen tablet technology to raise pupil learning outcomes research is needed that focuses not only on pupil learning outcomes, but also on critical aspects of implementation, such as teachers' use of and attitudes toward tablet technology and the embedding of tablet technology within the country's education system. These studies are essential in expanding our understanding of how touch screen tablet technology may help to reduce the challenges of underachievement in low resourced contexts. Studying in detail a country like Malawi, with all the challenges in its education system, presents an opportunity to illuminate the salient features of a successful digital integration within an education system in a low resourced country. Comparing the implementation of this technology across countries that have considerably different education systems, such as Malawi and the UK, enables generic features of implementation to be differentiated from country-specific factors. These generic and specific factors should then be used to test and successfully implement a theory of change.

Solar-powered touch screen tablet technology could prove to be a successful method for raising educational attainment in developing countries, offering sustainability and equality as countries strive to meet the 2030 objectives. However, the crucial factors of training teachers to use the technology effectively, management and resourcing of such large-scale projects, and the embedding of technology within education systems all need to be successfully addressed.

## Author contributions

All individuals listed as authors of this opinion piece have: Contributed substantially to the conception and design of the work; Drafted the work or revised it critically for important intellectual content; Have given final approval of the version to be published; and Agree to be accountable for all aspects of the work in ensuring that questions related to the accuracy or integrity of any part of the work are appropriately investigated and resolved.

## Funding

This work was supported by funding from Voluntary Service Overseas (Grant: MWI-14/0019 Unlocking Talent through Technology: Improving Learning Outcomes of Primary School Children in Malawi).

### Conflict of interest statement

The authors declare that the research was conducted in the absence of any commercial or financial relationships that could be construed as a potential conflict of interest.
